# *Radix natalensis*: the effect of *Fasciola hepatica* infection on the reproductive activity of the snail

**DOI:** 10.1051/parasite/2014026

**Published:** 2014-05-29

**Authors:** Yasser Dar, Philippe Vignoles, Daniel Rondelaud, Gilles Dreyfuss

**Affiliations:** 1 Department of Zoology, Faculty of Science, University of Tanta 31111 Tanta Egypt; 2 INSERM 1094, Faculties of Medicine and Pharmacy 87025 Limoges France

**Keywords:** Egg, Egg-laying, *Fasciola hepatica*, *Radix natalensis*, Snail infection

## Abstract

Experimental infections of Egyptian *Radix natalensis* (shell height at miracidial exposure: 4 mm) with a French isolate of *Fasciola hepatica* were carried out under laboratory conditions at 22 °C to specify the characteristics and follow the dynamics of their egg-laying. Controls constituted unexposed *R. natalensis* of the same size. No significant difference between controls and the uninfected snails of the exposed group was noted, whatever the parameter considered. In controls and exposed snails, the dates of the first egg masses were close to each other (56.4–65.3 days). In contrast, the life span of snails and the length of the egg-laying period were significantly shorter and egg production was significantly lower in infected *R. natalensis* than in controls and uninfected snails. In infected *R. natalensis*, but without cercarial shedding (NCS snails), egg production was irregular throughout the egg-laying period. In cercarial-shedding (CS) snails, the first egg masses were laid before the first cercarial emergence (at a mean of 56 days and 67 days, respectively). Thereafter, egg mass production of CS snails was irregular up to day 72 of the experiment, stopped during the following two weeks and started again after day 88 for a single snail. In conclusion, the *F. hepatica* infection of *R. natalensis* reduced the reproductive activity in both NCS and CS snails. The pattern noted for egg production in infected *R. natalensis* seems to be species-specific because of the high shell size of this lymnaeid and its role as an atypical intermediate host in the life cycle of the parasite.

## Abstract (fr)

Des infestations expérimentales de *Radix natalensis* provenant d’Égypte (hauteur de la coquille lors de l’exposition miracidienne, 4 mm) avec un isolat français de *Fasciola hepatica* ont été réalisées dans les conditions du laboratoire à 22 °C afin de préciser les caractéristiques des pontes et suivre leur dynamique. Des témoins ont été constitués avec des *R. natalensis* de même taille, mais non exposés au parasite. Aucune différence significative entre les témoins et les mollusques non infestés du groupe exposé n’a été relevée, quel que soit le paramètre considéré. Chez les témoins et les mollusques exposés, les dates des premières pontes sont proches les unes des autres (56,4–65,3 jours). Par contre, la durée de vie des limnées, la durée de la période de ponte et la production des œufs sont significativement plus faibles chez les *R. natalensis* infestés que chez les témoins et les mollusques non infestés. Chez les *R. natalensis* infestés mais sans émission cercariennes (limnées NEC), la production des œufs est irrégulière tout au long de la période de ponte. Chez les mollusques émettant des cercaires (limnées EC), les premières pontes ont été déposées avant les premières émissions cercariennes (respectivement au 56^ème^ jour et au 67^ème^ en moyenne). Par la suite, la production des pontes est irrégulière jusqu’au 72^ème^ jour de l’expérience avant de s’arrêter pendant les deux semaines suivantes et de reprendre au 88^ème^ jour chez un seul mollusque. En conclusion, l’infestation de *R. natalensis* par *F. hepatica* réduit l’activité reproductrice des limnées EC et NEC. La distribution des pontes chez les *R. natalensis* infestés semble être spécifique à l’espèce à cause de la grande hauteur de la coquille de cette limnée et de son rôle comme hôte intermédiaire atypique dans le cycle de vie du parasite.

## Introduction

The development of *Fasciola hepatica* within its intermediate host causes parasitic castration of the snail [7]. In *Galba truncatula*, the destruction of the gonad can be total in one-third of snails if a single or several rediae penetrate this gland and develop until they have ingested the whole contents [11, 17]. In the other two-thirds, the larval forms of *F. hepatica* only surround the gonad and cause its temporary atrophy, which induces a loss of the snail’s reproductive potential [12]. The number of eggs laid by *G. truncatula* progressively decreased until the fourth week postinfection at 20 °C, was stopped up to the eighth week, and increased later but only in single-miracidium infections of the snail [5]. Apart from some slight differences in the chronology, this last mode of parasitic castration was also observed by our team in snails infected with *Haplometra cylindracea* or *Paramphistomum daubneyi* [12].

In the juveniles of six European species of lymnaeids exposed to *F. hepatica* miracidia in the two weeks after their hatching, the destruction of the gonad was severe (the male line degenerated) and the gland did not develop thereafter [13–15]. As most European lymnaeids other than *G. truncatula* can sustain the larval development of *F. hepatica* when they are exposed to miracidia in their first days of life [1], it is difficult to determine changes which occur in the gonads of these snail species when they are infected at the preadult or adult stage. Recently, an Egyptian population of *Radix natalensis* was reported by Dar et al. [3] as an experimental intermediate host of *F. hepatica* when preadult snails were exposed to a French isolate of miracidia. In view of this last finding, the following two questions arose: was the egg production of *R. natalensis* affected when this lymnaeid was infected with *F. hepatica*? Was egg-laying of cercarial-shedding snails similar to that of individuals containing rediae and free cercariae but without shedding? To answer these questions, an experiment was carried out in the laboratory to study egg deposits in *R. natalensis* infected with *F. hepatica* and the results were compared with those from uninfected controls.

## Materials and methods

### Snails and parasite

The population of *R. natalensis* was living in an irrigation canal near the El-Mansouria River, Giza governorate, Egypt. A total of 150 adult snails were collected from this habitat and were transported to the laboratory before being placed in different types of covered aquaria with five snails per litre of permanently oxygenated spring water. These aquaria were kept in constant laboratory conditions: temperature, 22 ± 1 °C; light/dark period, 12 h/12 h. The dissolved calcium concentration in the spring water was 35 mg/L. Snails fed on pesticide-free leaves of fresh lettuce ad libitum and the spring water in the aquaria was changed weekly. Egg masses laid by these snails were collected using a fine forceps and were placed in small rearing aquaria. Newly hatched snails fed on finely powdered dry lettuce and those which attained 4 mm in shell height were used for experimental infections.

Eggs of *F. hepatica* were obtained from the gall bladders of infected cattle slaughtered at the abattoir of Limoges, department of Haute Vienne, central France. These eggs were washed several times with dechlorinated tap water and were incubated in the dark at 20 °C for 20 days. After this incubation period, the eggs were exposed to artificial light to enhance the emergence of miracidia.

### Experimental protocol

Fifty snails, measuring 4 ± 0.1 mm in height, were individually subjected twice to bimiracidial infections (2 + 2 miracidia/snail) with an interval of 4 h between the two exposures. The miracidial sequence used for snail infection came from the method adopted by Dar et al. [3, 4] for infection of Egyptian *R. natalensis* with a French isolate of *F. hepatica*. Another fifty snails of the same size were not exposed to infection and were used as a control group. Both exposed and control snails were then raised in the laboratory under constant conditions like their parents. The day of exposure was considered here as the onset of the experiment. After 4 weeks postexposure (p.e.), each surviving snail was placed in a 50-mm numbered Petri dish with 10 mL of spring water and a piece of fresh lettuce. The Petri dishes were examined daily to detect egg masses laid by the snails and/or cercariae which exited from infected snails. At the death of surviving snails, those which had not shed cercariae during the patent period were dissected under a stereomicroscope to detect the presence or absence of *F. hepatica* larval stages within their tissues and classify these cadavers into two subgroups: individuals containing rediae and free cercariae but without cercarial shedding (NCS snails), and those which were uninfected. Three subgroups: cercarial-shedding (CS) individuals, NCS, and uninfected snails were thus defined in the exposed group.

### Data analysis

The first five parameters were the number of surviving snails at week 4 p.e., their shell height at week 8, their life span, the time between the onset of the experiment and the first egg-laying, and the length of this oviposition period. The total number of eggs laid by each surviving snail during the experiment, the quantity of egg masses, the number of eggs per mass, and that of eggs per snail and per day were also taken into account. In order to specify potential relationships between the egg-laying period and cercarial shedding in the CS snails, the date of the first cercarial shedding and the length of the patent period were also considered.

Individual values recorded for the shell height of snails, their life span, the time between the onset of the experiment and the first egg-laying, the length of this oviposition period, and the numbers of egg masses and eggs were averaged and standard deviations were calculated taking into account the snail group or subgroup. One-way ANOVA coupled with the Tukey HSD test, the χ^2^ test, or the Kruskall-Wallis test were used to establish levels of statistical significance. All the analyses were performed using the Statview 5.0 software (SAS Institute Inc., Cary, NC, USA).

## Results

At week 4 p.e., the survival rate was 96% (48/50) in controls and 90.0% (45/50) in the exposed group. However, the differences between these survival rates were not significant.

### Characteristics of egg-laying

Table 1 gives the results noted in controls and the three categories of exposed snails. At week 8 p.e., the mean shell heights did not show any significant differences. The life spans of CS and NCS snails were significantly shorter (*H* = 20.42, *p* < 0.001) than those found in controls and the uninfected snails. No significant differences between the life spans of controls and uninfected snails, or between those of CS and NCS snails were noted. In controls and the three categories of exposed snails, the dates of the first egg masses were close to each other (56.4–63.5 days) so that differences between these mean values were not significant. The length of the egg-laying period was significantly shorter (*H* = 17.49, *p* < 0.001) in the CS and NCS categories than that of the other snails. As for the life span of snails, the differences between the lengths of egg-laying of controls and uninfected snails, or between those of NCS and CS snails were not significant. In controls and uninfected snails, the number of egg masses and the total number of eggs per snail were significantly higher (*F* = 17.5, *p* < 0.001; *F* = 10.67, *P* < 0.001, respectively) than those produced by the CS and NCS snails. The number of eggs per egg mass was significantly lower (*H* = 21.15, *p* < 0.001) in CS snails than in controls and the other two categories.

**Table 1.** Egg-laying of *Radix natalensis* (Egypt) subjected to two successive exposures (2 miracidia per snail in each case) with *Fasciola hepatica* (France). Each group consisted of 50 snails at the onset of the experiment. CS snails, cercarial-shedding snails; *n*, total number of surviving snails at week 4; NCS snails, snails containing free cercariae but without shedding.

Parameters	Unexposed controls (*n* = 48)	Exposed group (*n* = 45)		
		Uninfected snails	NCS snails	CS snails
Number of snails at week 4	48	21	13	11
Shell height at week 8 (mm)*	6.7 ± 1.1	7 ± 1.6	7.3 ± 2.7	7.2 ± 1.1
Life span of snails (days)*	138.2 ± 8.3	135.4 ± 23	93.7 ± 17.3	92.0 ± 7.6
Time between exposure and the first egg-laying (days)*	63.5 ± 9.3	65.3 ± 17.0	61.9 ± 29.2	56.4 ± 7.8
Length of the egg-laying period (days)*	74.7 ± 4.0	70.1 ± 19.5	31.8 ± 21.7	35.6 ± 7.5
Total number of egg masses*	24.9 ± 5.8	21.1 ± 7.5	9.2 ± 6.2	4.4 ± 2.1
Total number of eggs per surviving snail*	281.5 ± 108.9	237.4 ± 109.6	94.0 ± 81.5	32.4 ± 23.1
Number of eggs per egg mass*	11.3 ± 6.8	11.5 ± 4.6	10.3 ± 4.7	6.8 ± 4.9

* Mean value ± S.D.

### Dynamics of egg-laying

**Figure 1.**
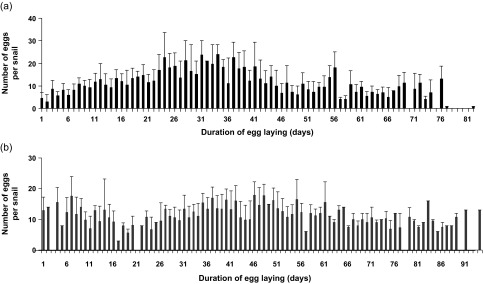
Numerical distribution of eggs per *Radix natalensis* and per day of the egg-laying period in unexposed controls (a) and the uninfected snails of the exposed group (b).

As no significant difference in the total number of eggs per *R. natalensis* was noted between controls and the uninfected snails, the pattern of eggs produced per snail, and per day was nearly the same (Figs. 1a and 1b). The mean number of eggs gradually increased until peaks at days 31 and 34 of the egg-laying period (24 eggs per snail and per day) for controls, and at days 46 and 48 (17.7 eggs per snail and per day) for uninfected snails.

**Figure 2.**
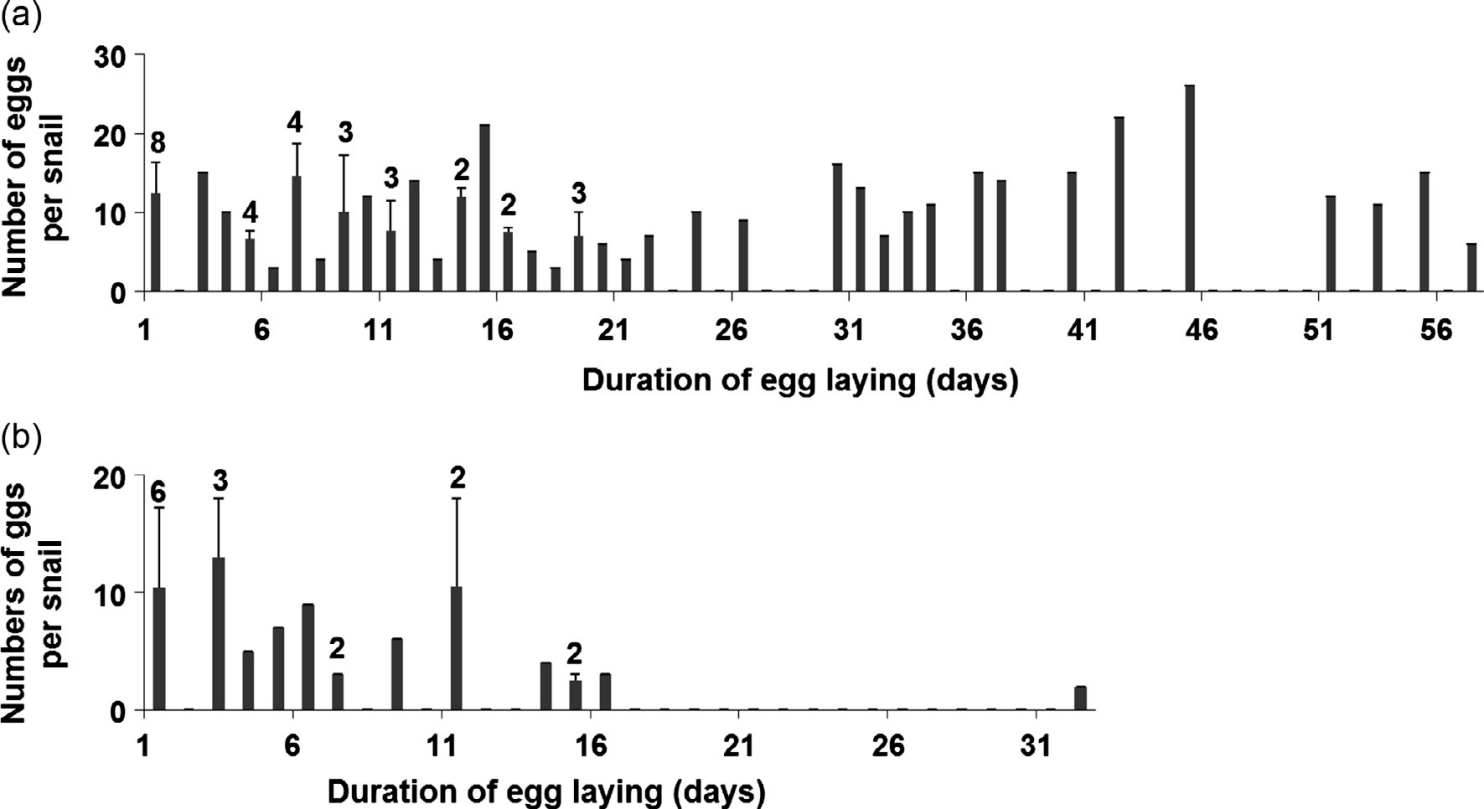
Numerical distribution of eggs per *Radix natalensis* and per day of the egg-laying period in the non-cercarial-shedding (2) and cercarial-shedding (2) snails of the exposed group. The figure on the top of each bar indicates the number of snails which laid eggs during this day (absence of figure: 1 snail only).

Figure 2 shows the dynamics of *R. natalensis* egg-laying over time in CS and NCS snails. In NCS snails (Fig. 2a), the values were irregularly distributed throughout the egg-laying period and most of them were due to the laying of a single snail for each day. In CS snails (Fig. 2b), the length of the period between exposure and the first cercarial shedding (prepatent period) was 67.6 ± 12.0 days, while the patent period until the snail’s death was 24.4 ± 10.1 days (data not shown).

**Figure 3.**
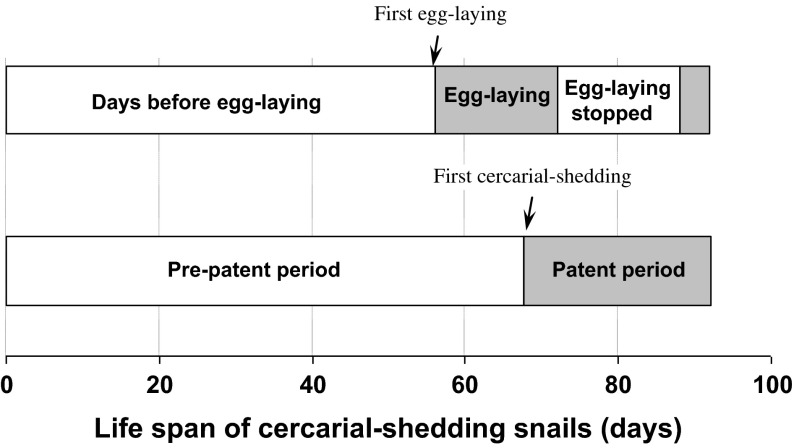
Mean durations of the egg-laying and patent periods for cercarial-shedding (CS) snails, with indication of dates for which egg-laying stopped and began again.

Comparison of the egg-laying period with the patent period (Fig. 3) demonstrated that the first egg masses were laid before the first cercarial shedding (at a mean of 56.4 and 67.6 days, respectively). Thereafter, egg-laying was irregular up to day 16 of the egg-laying period (until day 72 of the experiment) and was stopped thereafter during the other days of the patent period, in spite of an egg mass laid at day 32 of the egg-laying period (at day 88 of the experiment).

## Discussion

The present study demonstrates an irregular production of egg masses by NCS snails and a stopping of egg-laying from the 5th day of the patent period for CS snails. Although there were differences in the results noted for NCS and CS snails, the *F. hepatica* infection of *R. natalensis* had a clear effect by reducing egg production in the former snails and stopping it in the latter. These findings agree with the data reported by de Jong-Brink [10] and Thompson [16]. According to these authors, larval development of many trematodes had a negative impact on reproductive activity of the gonads and led to parasitic castration of the host snail. In the CS *R. natalensis*, the reduction in egg-laying and its stopping might be due (i) to the consumption of germinal cells by rediae and/or cercariae which developed in the visceral cavity of the snail, and/or (ii) to gonadal atrophy caused by pressure exerted by rediae and cercariae, as demonstrated in the model *G. truncatula*/*F. hepatica* [8, 11, 17]. However, the stopping of egg-laying in CS *R. natalensis* was shorter than that noted in the model *G. truncatula*/*F. hepatica*. According to Dreyfuss et al. [5], egg-laying of *G. truncatula* individually infected with one, two, or three miracidia per snail and raised at 20 °C was completely stopped from week 4 p.e. to the end of snail infection, apart from the one-miracidium group in which there was restoration of the snail’s reproductive activity from week 9. This discrepancy in the lengths of stopping periods of CS snails between the two *F. hepatica*-infected lymnaeids might mainly be due to the difference which exists between their shell sizes. As adult *R. natalensis* snails are higher and also wider than *G. truncatula* [2, 9], the former snails can offer more space and more nutrients for development of *F. hepatica* rediae [4]. As a consequence, developing daughter rediae grew at the expense of the digestive gland of snails before attacking their gonads. Contrary to CS snails, the irregular egg production noted in NCS snails from week 8 is more difficult to explain, taking into account the individual values of egg-laying obtained in this snail category. Indeed, these infected *R. natalensis* also contained free rediae and free cercariae like the corresponding CS snails, but the number of *F. hepatica* larval forms would be lower in the bodies of the former snails. An argument supporting this approach came from the report by Dreyfuss et al. [6]. According to these authors, the redial and cercarial production of *F. hepatica* in *G. truncatula* was lower when no cercarial shedding occurred. In the case of NCS *R. natalensis*, this lower number of larval forms would permit keeping the gonads in activity in several snails, thus explaining the irregular production of egg masses (Fig. 2a) from week 8 p.e. until snail death.

Restoration of reproductive activity in single-miracidium infections of snails was reported by Rondelaud and Barthe [11], Dreyfuss et al. [5], and Rondelaud et al. [12] in the model *G. truncatula*/*F. hepatica*. According to these authors, this restoration was only due to the reconstitution of the germinal epithelium at the end of the patent period when free cercariae present in the snail bodies were less numerous. In view of the single egg mass noted in the present study at day 32 of the egg-laying period (at day 88 of the experiment), it is difficult to consider it as the result of a reconstituted gonad for the following two reasons: (i) this egg mass was laid by a single *R. natalensis* (out of 11 CS snails), and (ii) these preadult snails were subjected twice to bimiracidial exposures so that numerous developing rediae (from 27.6 to 61 rediae per snail according to the number of developing sporocysts [4]) might invade the snail gonads or exert a pressure on them, thus leading to the interruption of gametogenesis. In NCS *R. natalensis*, there was a continuous pattern of egg-laying with an irregular distribution of egg masses during this period. If the hypothesis mentioned above for NCS snails is valid, egg masses deposited by these individuals during the egg-laying period would be due to a normal activity of snail gonads so that reconstitution of these glands would not occur in this category of infected snails.

In conclusion, the *F. hepatica* infection of *R. natalensis* reduced the reproductive activity in both NCS and CS snails. As the characteristics of egg-laying production in these infected snails were different from those reported for *G. truncatula*, the pattern noted in the former lymnaeid seemed to be species-specific because of the high shell size of *R. natalensis* and its role as an atypical intermediate host in the life cycle of the parasite [3, 4].
